# The role of financial distress as a patient-relevant endpoint in early benefit assessments for oncology drugs: a mixed-methods document analysis

**DOI:** 10.1017/S0266462326103626

**Published:** 2026-03-24

**Authors:** Sophie Pauge, John Andrew Grosser, Birthe Aufenberg, Wolfgang Greiner

**Affiliations:** School of Public Health, Department for Health Economics and Health Management, Bielefeld University, Germany

**Keywords:** quality of life, patient reported outcome measure, neoplasms, Germany, health technology assessment

## Abstract

**Objectives:**

The importance of Financial Distress (FD) for German cancer patients is rising and data on FD is increasingly being collected in clinical trials. However, the role of FD in German early benefit assessments (EBAs) remains unclear. We systematically analyze the role of FD in EBAs for oncology drugs in Germany by investigating how often and for what reasons FD is excluded for EBA by pharmaceutical companies, how often and in which ways FD is referenced in scientific assessments by German HTA authorities (IQWiG and G-BA), and how FD influences added benefit decisions.

**Methods:**

Our analysis covered all completed, currently valid German EBAs of oncology drugs from 2011 to 2024. We calculated exclusion rates, reference rates and benefit decisions, stratified by drug type, FD results, exclusion and reference status. In qualitative analyses, we inductively categorized reasons for FD exclusion and types of FD references.

**Results:**

FD currently plays at most a subordinated role for German EBAs across all involved stakeholders. Almost half of dossier submissions excluded FD from EBA, even though data on FD was collected. The IQWiG referenced FD in only 25 percent of their scientific assessments. Furthermore, the G-BA referenced FD in only three out of 215 justifications of resolution. HTA authorities have divergent and inconsistent approaches to FD.

**Conclusions:**

German HTA authorities should strengthen the role of patient-reported outcomes and provide clearer methodological guidance for integrating psychosocial dimensions such as FD. Future research should focus on refining measurement strategies to better capture the multidimensional nature of FD.

## Introduction

Recent advances in oncology have increased cancer incidence and prolonged survival, making cancer increasingly resemble a chronic disease (1). Oncological Health Technology Assessments (HTAs) should therefore focus not only on traditional clinical outcomes (e.g., survival, disease progression), but also on patient-reported outcomes (PROs) reflecting patients’ own perceptions of the disease and treatment (2–4).

In particular, cancer can have significant psychosocial consequences (5–7), including individual cancer-related financial distress (FD), an outcome that has been increasingly discussed for patients in publicly funded healthcare systems (8). For instance, a recent study from Germany demonstrated that cancer patients face significant reductions in their income and high out-of-pocket costs, with almost half of patients experiencing subjective distress (9).

FD refers to a multidimensional concept that includes both objective financial burdens, including direct medical costs (e.g., medications or therapies), direct non-medical costs (e.g., household- or work-related costs), and indirect costs (e.g., income loss), as well as subjective FD.

Subjective FD covers three domains: (i) material conditions, (ii) psychosocial responses, and (iii) behavioral coping (10;11). Subjective FD does not only result from objective FD as well as the anticipation of it, but also to patients’ individual response to objective FD (11;12). In Germany, subjective FD has recently been more precisely defined to encompass both an individual level, such as financial worries and dissatisfaction across various life domains, and an institutional level, referring to challenging experiences of cancer patients in interactions with authorities, benefit providers, and social services. Based on this conceptualization, a multidimensional instrument was developed; its validation study demonstrated a significant impact of subjective FD on health-related quality of life (HRQoL), anxiety and depression (9). These findings underscore the importance of assessing both objective and subjective FD.

However, in international clinical trials of oncology drugs, FD is only rarely assessed and, if so, it is primarily measured using a single item of the EORTC-QLQ-C30 questionnaire, which measures HRQoL in cancer patients. The self-administered 30-item instrument compromises five functional scales, three symptom scales, a global health status/QoL scale, and six single items. FD is one such single item, asking patients whether their physical condition or medical treatment has caused financial difficulties during the past week (Item 28) (13). This question reflects the material component of subjective FD but neglects the multidimensionality of FD. Furthermore, the short recall time might also underestimate the prevalence of FD.

German HTAs regularly accept EORTC-QLQ-C30 scales as demonstrating benefits in terms of morbidity (symptoms) and QoL (14), which, alongside mortality, are legally defined as patient-relevant endpoints (§ 35 Abs. 1b SGB V). Since the introduction of Germany’s Act on the Reform of the Market for Medicinal Products (AMNOG) in 2011, innovative drugs are assessed within an early benefit assessment (EBA) by the Federal Joint Committee (G-BA) after market entry to determine the added benefit of a drug compared to the standard of care. Orphan drugs (ODs) represent an exception, as their added benefit is automatically considered established upon European Medical Agency (EMA) approval; however, this OD status applies only until the drug reaches an annual turnover of <30 Mio Euro (before November 2022: <50 Mio Euro).

For EBAs, the pharmaceutical company (PC) submits a dossier that contains evidence on efficacy, safety, and patient-relevant endpoints from the pivotal clinical trials. For most drugs, the G-BA commissions the Institute for Quality and Efficiency in Health Care (IQWiG) to conduct the scientific assessment of the dossier and to make a recommendation for the quantification of the added benefit. For ODs with an annual turnover of <30 Mio Euro, however, the G-BA typically performs the scientific assessment itself. After this assessment, G-BA plenum makes the final decision on the added benefit of a drug by simple majority of its 13 voting members (the independent chair and representatives of statutory health insurers, physicians, and hospitals). In doing so, the G-BA considers the scientific assessment, the PC’s dossier, written statements, oral hearing contributions, and its own appraisal of clinical relevance, subgroup differentiation, certainty of evidence, and applicability to German routine care. This G-BA decision (called *resolution*) on the added benefit rating serves as the foundation for reimbursement negotiations between the National Association of Statutory Health Insurance Funds (GKV-SV) and the PC. Additionally, G-BA provides a written justification of each resolution.

The importance of FD for German cancer patients is rising and data on FD are increasingly being collected in clinical trials. However, the role of FD in the German EBA process remains unclear. Therefore, this paper aims to systematically analyze the role of FD (as measured by the EORTC-QLQ-C30) within the EBA process for oncology drugs in Germany by answering three main research questions (RQ):

- How often and for what reasons is FD excluded for EBA in the dossiers submitted by PCs?
- How often and in which ways is FD referenced in scientific assessments for EBA conducted by the IQWiG and G-BA?
- How do added benefit decisions by the G-BA depend on FD results, FD exclusion by PCs, and FD references by the IQWiG and G-BA?

## Methods

### Data selection

Our analysis covered all completed German EBAs of oncology drugs from 01/Jan/2011 until 31/Dec/2024, including new and re-assessed drugs, launch indications, and line extensions. We only considered currently valid decisions, to avoid double-counting assessments superseded by later evaluations. EBAs were identified using the G-BA’s HTA database (15) and filtered for completed assessments and oncology. We screened all PCs dossiers of the identified EBAs to determine whether the EORTC-QLQ-C30 had been administered in the pivotal clinical trial. Only dossiers for which this was the case were retained for analysis.

### Data analysis

All information was aggregated at the level of the overall assessment (referred to as EBA), rather than across the sub-indications specified by the G-BA in the course of the EBA. For each EBA, data were extracted from three sources: (1) the PCs submitted dossier, (2) the scientific assessment performed by the IQWiG or G-BA, and (3) the justifications of resolutions by the G-BA. These documents were systematically reviewed to identify relevant parameters, including study characteristics such as the number of underlying sub-indications, OD status, and added benefit rating. The dossiers were also screened for reported results of EORTC-QLQ-C30 Item 28. The results for FD were categorized into “significant positive results,” which refers to significantly higher FD of the intervention compared to the comparator, and “no significant positive results,” which includes all other FD results. In cases where these results were unavailable in the dossier, we hand-searched publications on the underlying clinical trials for analyses of Item 28. If no results were identified, we categorized it as “no results available.”

To address RQ I (exclusion of FD from EBAs by PCs), we performed a quantitative and qualitative analysis. For the quantitative analysis, we calculated the FD exclusion rate as the proportion of dossier submissions in which PCs excluded FD from EBAs, either by failing to report their FD results or by reporting FD results but explicitly excluding these results from consideration for the EBA. We then analyzed whether there were significant differences in exclusion rates by drug status (OD versus non-OD) and by FD results (significant positive results versus no significant positive results versus no results available). For the qualitative analysis, we investigated PCs stated reasons for excluding FD from EBAs by inductively developing categories of reasons, that is, the categories were derived from the data itself rather than based on predefined assumptions about possible reasons for exclusion.

To address RQ II (references to FD in scientific assessments by HTA authorities), we performed separate quantitative and qualitative analyses for scientific assessments performed by the IQWiG and G-BA. For each HTA authority, we calculated the FD reference rate as the proportion of scientific assessments which contained a reference to FD. We then analyzed whether there were significant differences in reference rates by drug status (only for the IQWiG), FD results, and exclusion of FD by the PCs. For the qualitative analysis, we inductively coded the references to FD made by the IQWiG and G-BA in their scientific assessments.

To address RQ III (impact of FD on added benefit decisions), we performed a quantitative analysis. Here, we calculated the proportion of benefit decisions that yielded any added benefit (major, considerable, minor, or non-quantifiable benefits versus no proven benefit). To assess the impact of FD on benefit decisions, we then analyzed whether there were significant differences in this proportion by FD results, exclusion of FD by the PCs, and reference to FD in the IQWiG’s scientific assessment. Finally, we determined the number of written justifications of resolution which contained references to FD. Because this number was very small, we did not analyze these references qualitatively.

Descriptive statistics were calculated and reported as percentages. We calculated odds ratios (ORs) and p-values using Fisher’s exact test. Where appropriate, differences between groups were visualized using bar graphs. All statistical analyses were carried out in RStudio (2025) using the packages tidyverse (16), magrittr (17), lubridate (18), dplyr (19), readx1 (20) and ggplot2 (21). Qualitative analyses were performed in Excel. Codes were inductively developed by two authors (SP and BA); dissent was solved by consensus with a third author (JAG). Subsequently, we calculated the relative frequencies of these inductive codes.

## Results

Out of 452 EBAs screened, a total of 215 were included. All applied the EORTC-QLQ-C30 questionnaire in their pivotal trial. Of these, 23 percent (n = 50) concerned an OD. For 62 percent (n = 134), the G-BA granted an added benefit. Overall, the majority of ODs were granted an added benefit, although 48 percent (n = 24) obtained this designation solely on the basis of their OD status.

A statistically significant positive intervention effect on FD was found in 10 percent (n = 22) of EBAs, that is the innovative treatment led to lower FD in the corresponding clinical trials. For 20 percent (n = 44) of EBAs, no evidence on FD was available. Results on FD were comparable between ODs and non-ODs. For detailed study characteristics, see Table 1.

**Table 1.** Study characteristics of included EBAs for oncology drugs applying the EORTC-QLQ-C30

	Total (N = 215)	Non-OD (N = 165)	OD (N = 50)
*G-BA sub-indications (M; SD)*	1.60 (0.90)	1.72 (0.96)	1.26 (0.56)
*Benefit rating*			
Added benefit	134 (62.33)	89 (53.94)	45 (90.00)
Major	9 (4.19)	6 (3.64)	3 (6.00)
Considerable	56 (26.05)	42 (25.45)	14 (28.00)
Minor	37 (17.21)	33 (20.00)	4 (8.00)
Non-quantifiable	32 (14.88)	8 (4.85)	24 (48.00)
Not proven	81 (37.67)	76 (46.06)	5 (10.00)
*Results for Financial distress*			
Pos. sign. FD results*	22 (10.23)	16 (9.70)	6 (12.00)
No pos. sign. FD results	149 (69.31)	115 (69.09)	36 (70.00)
Neg. sign. FD results*	5 (2.33)	3 (1.82)	2 (4.00)
No sign. FD results	144 (66.98)	112 (67.27)	33 (66.00)
Results not available	44 (20.47)	35 (21.21)	9 (18.00)

EBA, early benefit assessment; FD, financial distress; G-BA, Federal Joint Committee; M; mean, N, number; OD, orphan drug; pos, positive; SD, standard derivation; sign, significant.

* *P* ≤ 0.05 significance level

### RQ I: Exclusion of FD from EBAs by PCs

**Figure 1.**
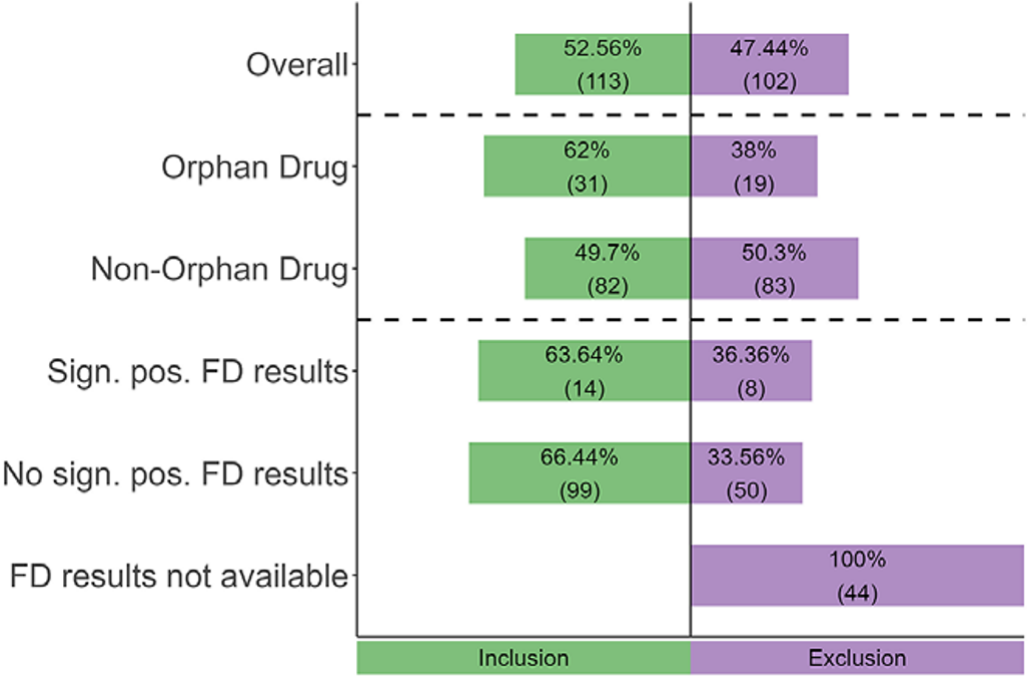
Exclusion of FD in EBAs by PCs. EBA, early benefit assessment; FD, financial distress; pos., positive; PC, pharmaceutical company; sign., significant.

Across all 215 dossiers, FD was excluded from the EBA by the PC in less than half of cases (see Figure 1). The exclusion rate was non-significantly lower for ODs than for non-ODs (OR = 0.61, 95 percent CI = 0.30–1.21, p = 0.147) and non-significantly higher when FD results were positive and significant compared to not (OR = 1.13, 95 percent CI: 0.38–3.12, p = 0.81). Dossiers for which no FD results from the underlying clinical trial were available anywhere could, by definition, only exclude FD results from EBAs.

Overall, more than half (60 percent) of PCs that excluded FD from EBAs provided an explanation for exclusion. In our qualitative analysis, we inductively developed three main categories of such explanations. First, for 69 percent of exclusions, PCs argued that FD is not a patient-relevant endpoint. In particular, PCs often explicitly argued that FD does not reflect symptoms relevant to morbidity. Second, 25 percent of exclusions referred to missing transferability of Item 28 to the German healthcare setting (as data were collected in other countries with differing healthcare systems and sociodemographics). Third, 6 percent of explanations argued that FD is not relevant in Germany (due to statutory health insurance).

### RQ II: References to FD in scientific assessments by IQWiG and G-BA

**Figure 2.**
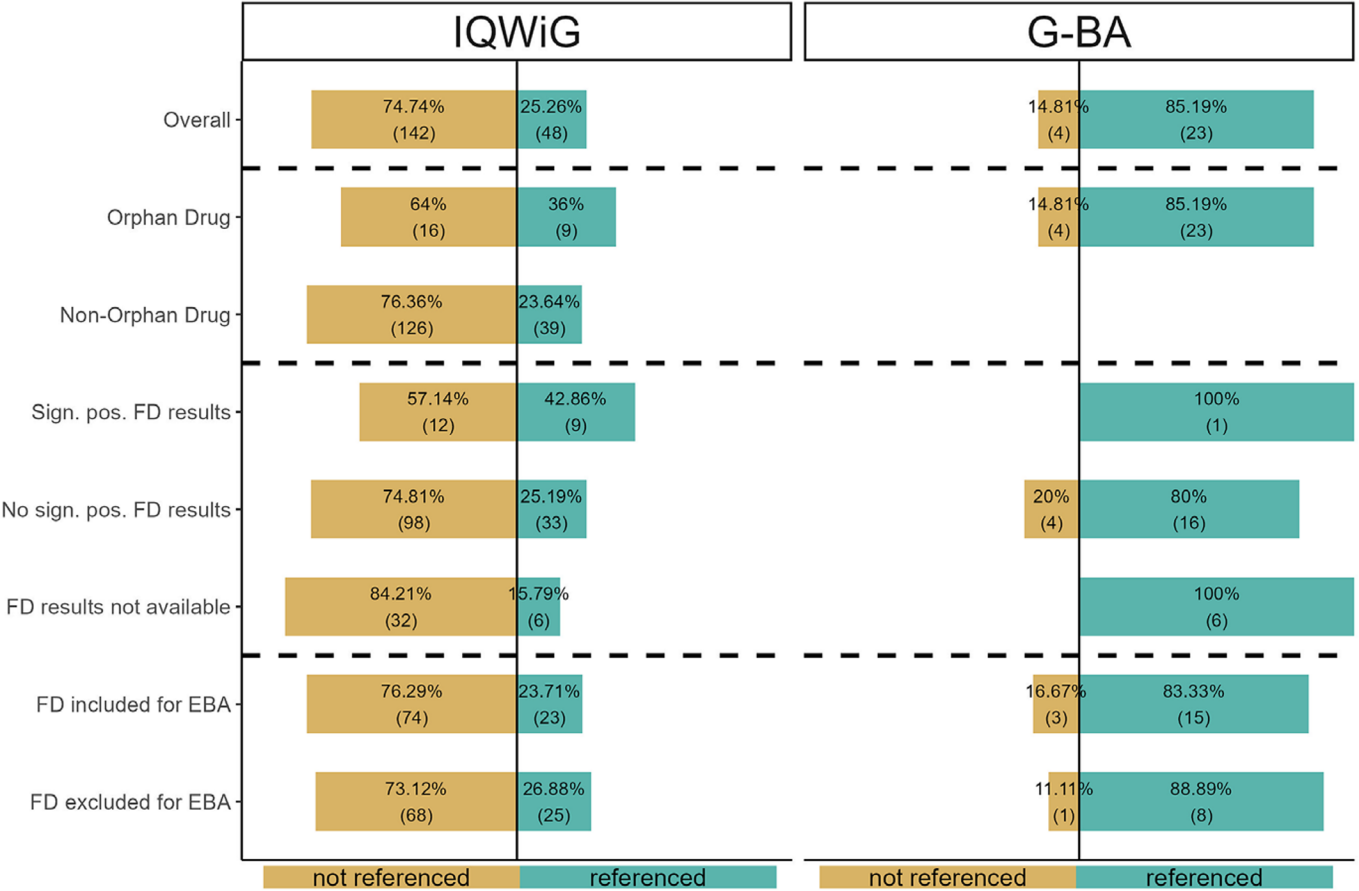
References to FD in scientific assessments by the IQWiG and G-BA. EBA, early benefit assessment; FD, financial distress; G-BA, Federal Joint Committee; IQWiG, Institute for Quality and Efficiency in Health Care; pos., positive; PC, pharmaceutical company; sign., significant.

There was a notable difference between HTA authorities in how often they referenced FD: less than 25 percent of IQWiG scientific assessments versus over 85 percent of G-BA scientific assessments (see Figure 2). For the IQWiG, this reference rate was non-significantly higher for ODs compared to non-ODs (OR = 1.81, 95 percent CI = 0.65–4.77, p = 0.22). For the G-BA, which only performs scientific assessments for certain ODs, no differences in reference rate by drug status could be assessed.

For the IQWiG, scientific assessments of dossiers with significant positive FD results referenced FD non-significantly more often than those without such results (OR = 2.20, 95 percent CI: 0.75–6.33, p = 0.12), while scientific assessments of dossiers with significant positive results referenced FD significantly more often than those without available FD results (OR = 3.89, 95 percent CI: 0.99–16.55, p = 0.03). The G-BA, however, referenced FD in all but four cases, all of which exhibited no significant positive FD results.

Finally, dossiers where the PC excluded FD from the EBA had a non-significantly slightly higher reference rate in scientific assessments by IQWiG (OR = 1.18, 95 percent CI = 0.58–2.40, p = 0.62) and G-BA (OR = 1.57, 95 percent CI = 0.10–94.29, p > 0.99) than dossiers where FD was not excluded.

For the IQWiG, our qualitative analysis yielded four main categories of references to FD: (1) In 55 percent of cases, the IQWiG merely mentioned FD as part of its description of the EORTC-QLQ-C30 questionnaire, (2) in 31 percent of cases, the IQWiG argued that FD is not a patient-relevant endpoint (usually in terms of morbidity or, occasionally, HRQoL); notably, in 53 percent of these cases, the IQWiG agreed with the exclusion of FD by the PC, while in the remaining 47 percent, it explicitly excluded FD even when the PC had not, (3) in 10 percent of cases, the IQWiG stated that FD results were not transferable to the German healthcare setting, and (4) in 4 percent of cases, FD was excluded from the EBA by the IQWiG without further explanation.

For the G-BA, we identified two categories of references: In 52 percent of cases, the G-BA argued that FD is not a patient-relevant endpoint, typically mentioning that FD does not reflect symptoms relevant to morbidity. Here, the G-BA followed the exclusion of FD by the PCs in 25 percent of cases, while in 75 percent it excluded FD independently. In the remaining 48 percent of cases, the G-BA merely mentioned FD as part of the EORTC-QLQ-C30 items without further explanation.

### RQ III: Impact of FD on added benefit decisions

**Figure 3.**
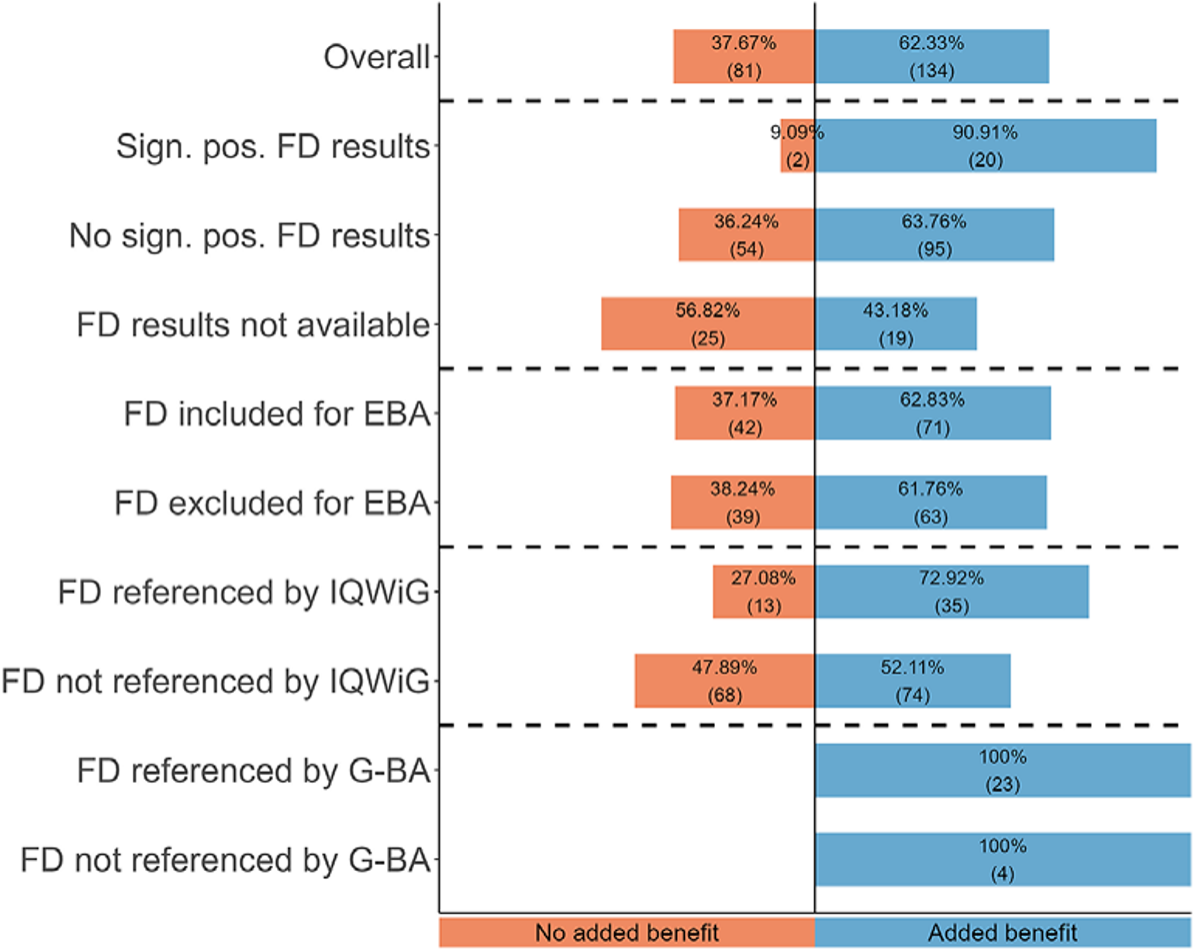
Added benefit decisions by the G-BA. EBA, early benefit assessment; FD, financial distress; G-BA, Federal Joint Committee; IQWiG, Institute for Quality and Efficiency in Health Care; pos., positive; PC, pharmaceutical company; sign., significant.

An added benefit was granted in 62 percent of cases (see Figure 3). Dossiers with significant positive FD results achieved an added benefit significantly more often than those with no significant FD results (OR = 5.64, 95 percent CI = 1.29–51.63, p = 0.01) and those with no FD results available (OR = 12.67, 95 percent CI = 2.57–125.24, p < 0.001). When FD was excluded for EBAs by the PCs, an added benefit was granted non-significantly less often than when PCs did not exclude FD (OR = 0.95, 95 percent CI = 0.53–1.72, p = 0.88). An added benefit was granted significantly more often when the IQWiG referenced FD in its scientific assessment than when it did not (OR = 2.46, 95 percent CI = 1.16–5.52, p = 0.01). In contrast, all drugs assessed by the G-BA received an added benefit due to their legal status as ODs.

Notably, the G-BA referenced FD in the justification of resolution in only three cases, even though the G-BA itself referenced FD in its own scientific assessment in most cases. In all three cases, the G-BA merely mentioned FD as part of the EORTC-QLQ-C30 questionnaire.

## Discussion

This study examined the role of FD, measured by the EORTC-QLQ-C30, within the German oncological EBA process. Our results indicate that FD currently plays at most a subordinated role for German EBAs across all involved stakeholders. However, our results also showed that significant positive FD results are associated with a significantly higher chance of receiving an added benefit.

Our results reveal major inconsistencies in how FD is addressed in EBAs, both between and within HTA authorities. While the G-BA referenced FD in 85 percent of their scientific assessments, the IQWiG did so in only 25 percent. Within the G-BA, the FD reference rate in scientific assessments were much higher (85 percent) than in justifications of resolution (1 percent).

What can explain these inconsistencies between and within HTA authorities? First, the G-BA and IQWiG differ in their mandate and objective. The IQWiG is mandated to scientifically evaluate PC’s dossiers and recommend a quantification of added benefit. The G-BA, in contrast, is responsible for the final decision on the added benefit, taking into account not only the scientific assessment, but further objectives relevant for the German healthcare context (see Introduction). Therefore, it is not surprising that the determined level of added benefit often differed between IQWiG and G-BA (22;23).

Secondly, our results reflect inconsistent approaches within HTA authorities, indicating methodological inconsistencies concerning FD. These inconsistencies may have influenced the approaches taken by the PCs, as the absence of consistent guidelines complicates the adequate reporting of FD in the dossier submission. This is illustrated by our analysis, where nearly half of all dossiers excluded FD from the EBA. This finding highlights a clear need for explicit and harmonized methodological guidance on how FD should be reported, analyzed, and interpreted in EBAs. Thus, the involved HTA authorities (G-BA and IQWiG) should coordinate and align their methodological requirements for the instrument to increase consistency across and within their scientific assessments.

Lastly, PCs and the IQWiG directly questioned the lack of transferability of FD results to the German healthcare system and the relevance of FD as a concept to German cancer patients. These attitudes might contribute to heterogeneous approaches within and between HTA authorities regarding how FD is considered, weighted, or even excluded in the scientific assessments and G-BA resolutions. Such attitudes appear questionable, however, in light of several studies demonstrating that FD is prevalent among German cancer patients and is significantly associated with higher levels of depression and anxiety as well as with a deterioration of HRQoL (9;24–28). Given its substantial impact on patients, HTA authorities should recognize FD as an important dimension in oncology HTA assessments and make efforts to promote a more holistic evaluation of FD in the EBA using a comprehensive instrument.

In Germany, PROs can be used either to capture symptoms under the morbidity endpoint or as part of HRQoL (29). The EORTC-QLQ-C30 covers both morbidity and HRQoL, necessitating the allocation of its items and scales to the respective endpoints (14). But, if FD is to be considered in EBA, should it be considered a morbidity endpoint or an HRQoL endpoint?

The IQWiG generally argued that FD is not a morbidity endpoint, but did not consider whether it could be considered as an HRQoL endpoint. This possibility was discussed by the IQWiG in only a small number of cases. The G-BA, on the other hand, consistently argued that FD is not a morbidity endpoint, never considering whether it could be considered an HRQoL endpoint.

The conceptual framework of the EORTC-QLQ-C30 defines HRQoL more broadly, comprising functional status, physical symptoms, psychological distress, social interaction, financial impact, perceived health status, and overall QoL (5). Within this framework, symptoms form one component of QoL, while FD is explicitly considered as another, separate dimension. The IQWiG, however, applies a narrower interpretation, in which only global QoL and the subscales for physical, emotional, cognitive, social, and role functioning are counted as HRQoL, while symptoms are classified under morbidity (14) and FD is, generally, excluded altogether.

In any case, the EORTC-QLQ-C30 as a whole has been validated as an instrument to measure quality of life in cancer patients. In its methods for EBAs, the IQWiG requires validated PRO instruments, explicitly referring to guidance which emphasizes that PROs must reliably capture the underlying concept they are intended to measure (29;30). This suggests that the EORTC-QLQ-C30, as a validated instrument, should be evaluated in line with its conceptual framework. Excluding individual components such as FD from consideration undermines the integrity of the instrument and risks a selective application of evidence. Therefore, regardless of whether FD is considered to be a morbidity or an HRQoL endpoint, FD results from the EORTC-QLQ-C30 should be included in EBA. Since both HTA authorities did not accept FD as a measure for symptoms of the morbidity endpoint, an interpretation of FD as part of HRQoL appears methodologically more consistent with the instrument’s conceptual framework.

The role of FD in HTA has so far received little systematic attention. One of the first contributions in this area proposed considering individual FD in economic evaluations (31). In Germany, however, economic evaluations play a marginal role, as the focus of HTA is restricted to relative clinical benefit assessment. Thus, FD can only be captured indirectly under existing patient-relevant endpoints. While the importance of patients’ perspectives (via PROs) is acknowledged in German law, PROs still play a minor role in decisions about added benefit in oncology (32–34). This narrow focus risks overlooking psychosocial dimensions that are highly relevant for cancer as a chronic disease. To address this, the role of PROs in HTA must be strengthened overall. In the long term, this would allow FD to be meaningfully integrated into German EBA, contributing to a more patient-centered understanding of benefits beyond traditional endpoints.

However, since the EORTC-QLQ-C30 assesses FD with only a single-item measure referring to the past week, it may not adequately capture the multidimensional nature of FD (9;11;35). Considering FD’s demonstrated importance within the German healthcare setting and its impact on critical health outcomes for cancer patients, it may be necessary to further broaden and refine the scope of how FD is assessed and integrated into HTA processes in future research.

### Limitations

Our analysis relied on clinical trials using the EORTC QLQ-C30, which provided a broad evidence base due to its frequent use in German EBAs. However, FD was measured using a single item, potentially underestimating its prevalence and extent as a complex construct. In addition, internationally recruited and highly selected trial populations may limit the transferability of FD findings to routine care in the German healthcare system. Our analysis was aggregated at the level of EBAs rather than at the level of sub-indications used by the G-BA to determine added benefit. This broader perspective was necessary to enable hand-searching of FD results not reported in dossiers but potentially published elsewhere; however, it may have led to an overestimation of the impact of FD results on EBAs. While additional FD results identified by manual search provided valuable insights, they should be interpreted with caution, as publication bias is likely for the most recent and potentially unpublished findings. If no significance level was reported for FD results, we assumed no significant intervention effect, which may underestimate the proportion of significant effects. Finally, the observed association between positive FD outcomes and a higher likelihood of added benefit should be interpreted with caution, as FD may partly reflect the overall strength of the underlying trial evidence and the quality of the dossier submissions rather than exerting an independent effect. Other studies suggest that dossiers with comprehensive evidence, especially in terms of mortality and morbidity endpoints (34), are more likely to receive an added benefit by G-BA (36). The lack of adjustment for potential confounders and the automatic attribution of added benefit to ODs by law may have influenced the analysis of RQ III.

## Conclusion

This study demonstrates that FD, although frequently measured in clinical trials via the EORTC-QLQ-C30, remains inconsistently considered in German EBAs. Both the IQWiG and the G-BA have applied divergent and sometimes incoherent approaches, often underestimating the relevance of FD for German cancer patients. To ensure a more patient-centered evaluation of oncology innovations, several implications merit consideration: (i) greater alignment of methodological approaches to scientific assessment both between and within HTA authorities; (ii) systematic consideration of the FD item when the EORTC QLQ-C30 is used in EBA, given its relevance for German cancer patients and the need to ensure methodological rigor; (iii) explicit recognition of FD as a component of HRQoL; (iv) strengthening the role of PROs within German HTAs. Future research should focus on refining measurement strategies to better capture the multidimensional nature of FD and to integrate it adequately in EBA.
